# Healthcare providers perceptions regarding the presence of Birth Companion during childbirth at a tertiary care hospital in India

**DOI:** 10.1186/s12884-022-05327-1

**Published:** 2023-03-10

**Authors:** Tanvi Sarwal, Yamini Sarwal, Shakun Tyagi, Rakesh Sarwal

**Affiliations:** 1 All India Institute of Medical Sciences, Jodhpur, India; 2 Vardhman Mahavir Medical College and Safdarjung Hospital, New Delhi, India; 3 Maulana Azad Medical College, New Delhi, India; 4 National Minorities Development & Finance Corporation, New Delhi, India

**Keywords:** Birth Companion, Childbirth, Birthing, Respectful Maternity Care, Privacy, Healthcare Providers, COVID-19

## Abstract

**Background:**

Despite an increase in institutional births and a fall in maternal mortality, the satisfaction of women with their birthing experience in public health institutions is low. Birth Companion (BC) is an important part of the Labour Room Quality Improvement Initiative introduced by the Government of India in 2017. Despite mandates, its implementation has been unsatisfactory. Little is known about the perception of healthcare providers about BC.

**Methods:**

We conducted a facility-based, cross-sectional quantitative study with doctors and nurses in a tertiary care hospital in Delhi, India to gauge their awareness, perception and knowledge about BC. Following universal total population sampling, the participants were administered a questionnaire, which was completed by 96 of 115 serving doctors (response rate of 83%), and 55 of 105 serving nurses (response rate of 52%).

**Results:**

Most (93%) healthcare providers were aware of the concept of BC, WHO’s recommendation (83%) and Government’s instructions (68%) on BC during labour. A woman’s mother was the BC of choice (70%) closely followed by her husband (69%). Ninety-five percent of providers agreed that the presence of a BC during labour will be beneficial, in providing emotional support, boosting the woman’s confidence, providing comfort measures, helping in the early initiation of breastfeeding, reducing post-partum depression, humanizing labour, reducing the need for analgesia and increasing chances of spontaneous vaginal births. Yet, support for the introduction of BC in their hospital was low due to institutional barriers like overcrowding, lack of privacy, hospital policy, risk of infection; privacy issues and costs.

**Conclusions:**

Widespread adoption of the concept of BC would require, besides directives, a buy-in by the providers, and action on their suggestions. These include greater funding for hospitals, creating physical partitions to ensure privacy, sensitization and training of health providers and BC, incentivizing hospitals and birthing women, formulation of guidelines on BC, standards setting and a change in institutional culture.

**Supplementary Information:**

The online version contains supplementary material available at 10.1186/s12884-022-05327-1.

## Background

Skilled care in pregnancy and childbirth is considered necessary to improve birth outcomes [1]. Globally, a 31% increase (64% to 84%)​ [2] in skilled birth attendance during the period 2001–2021 was accompanied by a 38% decline (342 to 211 per 100,000 live births) in maternal mortality during 2000–2017 [3]. In India, the change has been dramatic with institutional births increasing 2.6 times (from 33.6% in 1998–99​ [4] to 88.6% in 2019–21 [5]) and the Maternal Mortality Ratio (MMR) dropping by three-quarters during this period from 398 to 99 [6].

Despite this progress, many women face disrespectful treatment from healthcare providers in the form of physical, verbal and emotional abuse, neglect and disrespect [7, 8] leading to a low satisfaction among women birthing in public health institutions [9, 10]. This also negatively influences maternal and newborn outcomes [11]. Fear of being alone in unfamiliar surroundings, in contrast to ‘safe and reassuring environment’ at home, compels some women to give birth at home [12, 13].

Respect for human rights includes the right to self-determination and privacy embodied in the idea of Respectful Maternity Care (RMC), which also includes a companion of choice throughout labour [14, 15]. Presence of a Birth Companion (BC) in labour is known to lead to a shorter duration of labour [16]. Continuous, one‐to‐one intrapartum support during labour can improve outcomes for women and infants, including spontaneous vaginal birth, shorter duration of labour, and less likelihood of caesarean birth, instrumental vaginal birth, use of any analgesia, low five‐minute Apgar score and negative childbirth experience [17]. WHO’s intrapartum guidelines recommend a “positive childbirth experience for all women undergoing labor, which includes a clinically and psychologically safe environment with continuity of practical and emotional support from a birth companion” [15]. WHO strongly advocates for the presence of a BC of choice during childbirth [18–20].

BC have been found to be feasible and well accepted by health providers and the birthing women in a pilot study in Tanzania [21]. Another cross-sectional study in Tanzania concluded that health providers are the gatekeepers of companionship, and their work environment influences providers’ allowance of companionship [22]. Concluding that the consultants often decide policy, a Sri Lankan study underlined the need to improve awareness of BC among the practitioners [23]. Hence, many countries like Sri Lanka,​ [24] Tanzania, [25] Brazil, [26] Kenya [27] have supported BC in their public facilities.

In India, despite the 2016 advisory of the health ministry permitting BC during childbirth in Public Health facilities,​ [28] only some states like Tamil Nadu (2002), [29, 30] Kerala,​ [31, 32] and Punjab [33] have supported BC in their public facilities. A Labour Room Quality Improvement Initiative (LaQshya)​ [34] was thus launched in 2017 in all Medical Colleges, District Hospitals, First Referral Units, and Community Health Centers to ‘fast-track’ the interventions for RMC through a system of certification and monetary incentive to institutions [35]. Though the original plan was to “achieve tangible results within 18 months”, only 27% of the Labour Rooms (410) and Maternity Operation Theatres (337) [36] of the 2805 identified facilities in the country [37] had obtained the National level certification by September, 2021.

Slow progress in the introduction of BC has been globally attributed to several factors. These include a lack of awareness among the healthcare providers of the benefits associated with the practice, concerns among the healthcare providers, health system factors, lack of physical space in the labour room and the volume of work [23]. Other factors include hospital policy, [38] embarrassment, fear of gossip and privacy concerns [39].

Little is known in India about the awareness or the perception of healthcare providers about BC, beyond a few studies [40]. We conducted this facility based study to fill this gap in evidence on the awareness, perception and knowledge among healthcare providers regarding BC. Our study objectives were:

1. To assess the level of awareness among the entire spectrum of caregivers in a tertiary care teaching hospital in India of the concept and benefits of a BC during labour and childbirth.
2. To understand their perception of the barriers to supporting the presence of BC during labour and childbirth in an institutional setting.
3. To gather suggestions from caregivers on how these barriers could be overcome.

## Methods

### Design

We conducted this facility based, cross-sectional study using quantitative survey methods. The rationale behind this study design was to collect data from a large pool of participants in a limited time period.

### Setting

The study was conducted in June and July of 2019 in the Department of Obstetrics & Gynecology, Lok Nayak Hospital, Maulana Azad Medical College, New Delhi, India, which is one of the largest medical institutes in the country. It hosts over 1500 childbirths per month. Around eight to ten women are in labour at any point of time, with two to three doctors posted in each shift, round the clock. The obstetrics ward has forty beds, while the labour room has fifteen beds. Women are triaged at the time of admission. Women in early labour (upto 4 cm of cervical dilatation) are admitted to the wards, and those in an advanced stage of labour are admitted directly to the labour room.

### Participants

The study population consisted of all the medical and nursing healthcare providers involved in providing institutional care during labour and birthing, including obstetric Consultants, Senior Residents, Post- Graduate doctors and all grades of nursing Staff.

Sampling involved "Universal total population sampling” [41] whereby all individuals belonging to the study population and present at the time of the study were approached for participation by sharing the Study Participant Information Sheet (Additional file, Annexure 1). This was done to eliminate any selection bias or sampling error. Those healthcare providers who expressed willingness to participate were given the Consent Form (Additional file, Annexure 2) for signature. These respondents were then handed over a printed questionnaire with a request to enter their response. Filled response sheets, along with consent forms, were personally collected from the participants. Since the PI was an Undergraduate student, her involvement in the interview and data collection could not have placed the participants under any coercion.. Of 115 doctors, 96 completed the questionnaire (response rate of 83%), while 55 of 105 nurses completed the questionnaire (response rates of 52%). With a sample of 151, our overall response rate was 69%. Most (95%) of the respondents were females.

### Ethical clearance

The Institutional Ethics Committee of Maulana Azad Medical College accorded clearance for the study (order No. F.17/IEC/MAMC/19/No. 125 Dated 27.5.2019). Prior to canvassing of the questionnaire, informed consent of the willing respondents was obtained in writing. Complete confidentiality of the participants and their responses was maintained by giving each respondent an ID number and not sharing any personal identifiers. Participants were given the choice to opt out of the study without any condition.

### Study instrument

Based on a thorough literature review, a 15 point study questionnaire was framed, and pre-tested on 10 subjects, before its finalization (Additional file, Annexure 3). The first three questions captured the socio-demographic characteristics and position of participants in the department. Questions 4–10 related to awareness of the concept and benefits of BC. Subsequent questions numbered 11–15 were on their perception of the barriers and suggestions.

### Data analysis

The information collected was entered in a Google form, and results downloaded as a Comma Separated Values (csv) file. Data were checked for consistency and completeness; data entry errors were spotted and corrected. Cleaned data were analyzed in Stata 8.0. Labels were assigned to variables. Ordinal variables were coded in a consistent, hierarchical manner with a score of one (1) denoting full awareness, highly beneficial or strong agreement.

Our questionnaire elicited responses on awareness, benefits, barriers, suggestions and applicability of the concept of BC in the hospital. Any systematic differences in outcomes by demographic variables or position in the department were ascertained.

The study data contained responses of participants to various questions – either as binary, or on an ordinal scale of an order of three or five. Since ordinal variables and differences between them are neither uniform nor can be assumed to be normally distributed, standard measurements methods like means, standard deviations and t-test were not suitable [42]. Instead, we used non-parametric tests [43] like Pearson Chi Square statistic, Kendall's tau-b, Kruskal’s gamma statistic and Kruskal–Wallis test that do not rely on the assumption of normal distribution. We also calculated the median and percentages. Fisher’s exact test was applied to tables with cell values less than five, Quantile regression was used for a similar reason. *P*-value of less than 0.05 was considered significant.

## Results

Two third of the respondents (64%) were doctors (10 consultants, 47 Post-Graduates, 39 residents), and the remainder were nurses (15 senior nurses and 40 staff nurses). The participants were aged 21 to 55 years, with a mean age of 31 years (Table 1).

**Table 1:** Demographic Characters of Respondents

Position	Numbers (%)	Mean age	Female	Male
Consultant	10 (7)	47	9	1
Post Graduate	47 (31)	26	42	5
Resident	39 (26)	29	37	2
Sr. Nurse	15 (10)	43	15	0
Staff Nurse	40 (26)	32	40	0
Total	151 (100)	31	143	8

### Awareness of birth companion among healthcare providers

Awareness of the concept of BC was high with 53% of respondents being fully aware and 40% being somewhat aware (overall: 93%). Similarly, awareness of WHO’s recommendation of the presence of BC during labour was high with 54% respondents being fully aware and 29% being partially aware (overall: 83%).

Median awareness for both questions was 1, denoting full awareness on a three point scale with 3 being not aware. Sub-group analysis revealed that consultants and staff nurses had the highest level of awareness (mean and median awareness was 1 and close to 1).

The awareness of BC and WHO’s recommendation were highly correlated with a Pearson Chi Square statistic of 55.6 with a *p*-value of < 0.001 denoting a high level of overlap of the awareness of the concept and the guidelines on it (Table 2).

**Table 2:** Awareness of BC vs WHO Recommendation Regarding Birth Companion

Q4. Are you aware of the concept of Birth Companion ?	Q5. Are you aware that the WHO recommends every woman to be accompanied by a Birth Companion ?			
	Fully Aware	Somewhat aware	Not aware	Total
Fully Aware	57	16	7	80
	(71.25)	(20)	(8.75)	(100)
	(69.51)	(36.36)	(28)	(52.98)
Somewhat Aware	25	27	9	61
	(40.98)	(44.26)	(14.75)	(100)
	(30.49)	(61.36)	(36)	(40.4)
Not Aware	0	1	9	10
	(0)	(10)	(90)	(100)
	(0)	(2.27)	(36)	(6.62)
Total	82	44	25	151
	(54.3)	(29.14)	(16.56)	(100)
	(100)	(100)	(100)	(100)

Pearson chi2(4) = 55.6358 Pr = 0.000

Cramér's V = 0.4292

gamma = 0.6271 ASE = 0.091

Kendall's tau-b = 0.4023 ASE = 0.072

Fisher's exact = 0.000

Similarly, Fisher’s exact test also showed dependence of the response to the two questions (*p* = 0.00). Kendall's tau-b coefficient to test the strength of the association of cross tabulations showed an Alpha’s Standard Error (ASE) of 0.072 denoting dependence of two sets of responses. Kruskal’s gamma statistic at 0.62 and ASE of 0.091 also similarly showed a material level of association.

On the question of awareness of the Government’s guideline on the presence of BC (Q.6), awareness levels were slightly lower with 39% being fully aware, while 29% were somewhat aware (overall awareness: 68%) and an overall median score of 2. Consultants were still highly aware of this stipulation, while senior nurses were least aware of it.

Of those who were fully aware of the concept of BC, 71% were also fully aware of the WHO’s recommendation (Table 2) and 56% were fully aware that Government of India advocates the presence of BC (Table 3). The association to the responses of awareness of the concept of BC and the Government’s advisory was significant with a Pearson Chi Square statistic (χ2) with 4 degrees of freedom of 29.6 (*p*-value: 0.00), Fisher’s exact test *p*-value of 0.00, Kendall's tau-b of 0.37 (ASE: 0.067), Kruskal’s gamma statistic at 0.58 and ASE of 0.091 (Table 3).

**Table 3:** Awareness of Birth Companion vs. that of Government Guidelines

Q4. Are you aware of the concept of birth Companion ?	Q6. Are you aware that the Government of India has recently advocated the presence of BC in all government hospitals ?			
	Fully Aware	Somewhat aware	Not aware	Total
	N			
	(Row, Column Percentages)			
Fully Aware	45	20	15	80
	(56.25)	(25)	(18.75)	(100)
	(76.27)	(45.45)	(31.25)	(52.98)
Somewhat Aware	13	23	25	61
	(21.31)	(37.7)	(40.98)	(100)
	(22.03)	(52.27)	(52.08)	(40.4)
Not aware	1	1	8	10
	(10)	(10)	(80)	(100)
	(1.69)	(2.27)	(16.67)	(6.62)
Total	59	44	48	151
	(39.07)	(29.14)	(31.79)	(100)
	(100)	(100)	(100)	(100)

Pearson chi2(4) = 29.6057 Pr = 0.000

likelihood-ratio chi2(4) = 29.5292 Pr = 0.000

Cramér's V = 0.3131

gamma = 0.5851 ASE = 0.091

Kendall's tau-b = 0.3780 ASE = 0.067

Fisher's exact = 0.000

Similarly, 62% of respondents were fully aware of WHO’s recommendation as well as the Government’s advisory; these responses were significantly correlated with a Pearson Chi Square statistic (χ2) with 4 degrees of freedom of 64.5 (*p*-value: 0.00), Fisher’s exact test *p*-value of 0.00, Kendall's tau-b of 0.56 (ASE: 0.054), Kruskal’s gamma statistic at 0.78 and ASE of 0.057 (Table 4).

**Table 4:** Awareness of WHO recommendation vs. Government Guidelines

Q5. Are you aware that the WHO recommends every woman to be accompanied by a Birth companion ?	Q6. Are you aware that the Government of India has recently advocated the presence of BC in all government hospitals ?			
	Fully Aware	Somewhat aware	Not aware	Total
	N			
	(Row, Column Percentages)			
Fully Aware	51	22	9	82
	(62.2)	(26.83)	(10.98)	(100)
	(86.44)	(50)	(18.75)	(54.3)
Somewhat Aware	7	19	18	44
	(15.91)	(43.18)	(40.91)	(100)
	(11.86)	(43.18)	(37.5)	(29.14)
Not Aware	1	3	21	25
	(4)	(12)	(84)	(100)
	(1.69)	(6.82)	(43.75)	(16.56)
Total	59	44	48	151
	(39.07)	(29.14)	(31.79)	(100)
	(100)	(100)	(100)	(100)

Pearson chi2(4) = 64.5458 Pr = 0.000

likelihood-ratio chi2(4) = 67.0192 Pr = 0.000

Cramér's V = 0.4623

gamma = 0.7883 ASE = 0.057

Kendall's tau-b = 0.5602 ASE = 0.054

Fisher's exact = 0.000

#### Preferred BC of choice

Respondents were asked to choose the preferred BC from a list, allowing multiple selections. A woman’s mother was the BC of choice (70%) closely followed by a husband (69%). Other preferences included sister (46%), nurse (43%), mother-in-law (34%), friend (30%) and doctor (28%).

#### Pre-requisites for a BC

The pre-requisite for being a BC were identified as wearing clean clothes (95%), not suffering from any communicable disease (91%), and staying with the woman throughout the process of labour (74%). Less than half of the respondents opted for a BC having gone through the process of labour (42%), or being a female relative (40%).

#### Perceived benefits of a BC

Respondents were then asked about their knowledge of the likely benefits of a BC Just over half of the respondents (51%) viewed BC to be highly beneficial; many (44%) felt it was somewhat beneficial (Table  5) with an overall median score of 1. Most Consultants, Post-Graduates and Senior Nurses opined it to be highly beneficial with a median score of 1. Residents and Staff Nurses were muted in their response and believed BC would be somewhat beneficial.

Almost all respondents (99%) were of the opinion that BC would provide emotional support and boost the woman’s confidence (Table 5). More than 90% of participants were of the opinion that the BC would provide comfort, spiritual support, increase satisfaction, help in early initiation of breastfeeding, be an advocate for a woman’s wishes, and reduce postpartum depression. A majority of the respondents thought that BC would lead to humanization of labour, reduce the need for analgesia, unnecessary cesarean sections, and instrumental childbirth, reduce the workload for the hospital staff, increase spontaneous vaginal births and help in enabling a woman birth in her position of choice. Half the respondents thought that it would lead to a higher newborn Apgar score. Some respondents expected BC to lead to a shorter duration of labour (38%), or reduced intra-partum bleeding (36%) or increased use of partograph (26%, Table 5).

**Table 5:** Perceived benefits of a Birth Companion

Variable	N	Agree	%
10.10 Emotional support	151	149	99%
10.14 Boosting woman’s confidence	151	148	98%
10.11 Comfort measures – soothing touch, massage	151	144	95%
10.16 Increased satisfaction by women	151	142	94%
10.15 Spiritual support	151	141	93%
10.18 Early initiation of breastfeeding	151	141	93%
10.12 Helping the woman to advocate her wish to others	151	140	93%
10.20 Reduced post-partum depression	151	137	91%
10.6 Humanization of labour	151	126	83%
10.2 Reduced need for analgesia	151	106	70%
10.3 Increased spontaneous vaginal births	151	104	69%
10.5 Reduced incidence of unnecessary cessarian sections	150	98	65%
10.4 Reduced need for instrumental childbirth	151	92	61%
10.7 Reduced workload for hospital staff	151	86	57%
10.13 Childbirth in birth position of choice	151	82	54%
10.19 Higher newborn Apgar score	151	75	50%
10.8 Avoid frequent vaginal examination	151	60	40%
10.1 Shorter duration of labour	151	58	38%
10.9 Reduced intrapartum bleeding	151	54	36%
10.17 Increased use of partograph	151	39	26%

Though most respondents thought that BC could be “somewhat beneficial” in dealing with high risk pregnancies, Consultants viewed BC to be “highly beneficial” in this regard.

#### Readiness for introducing BC in the hospital

**Fig. 1:**
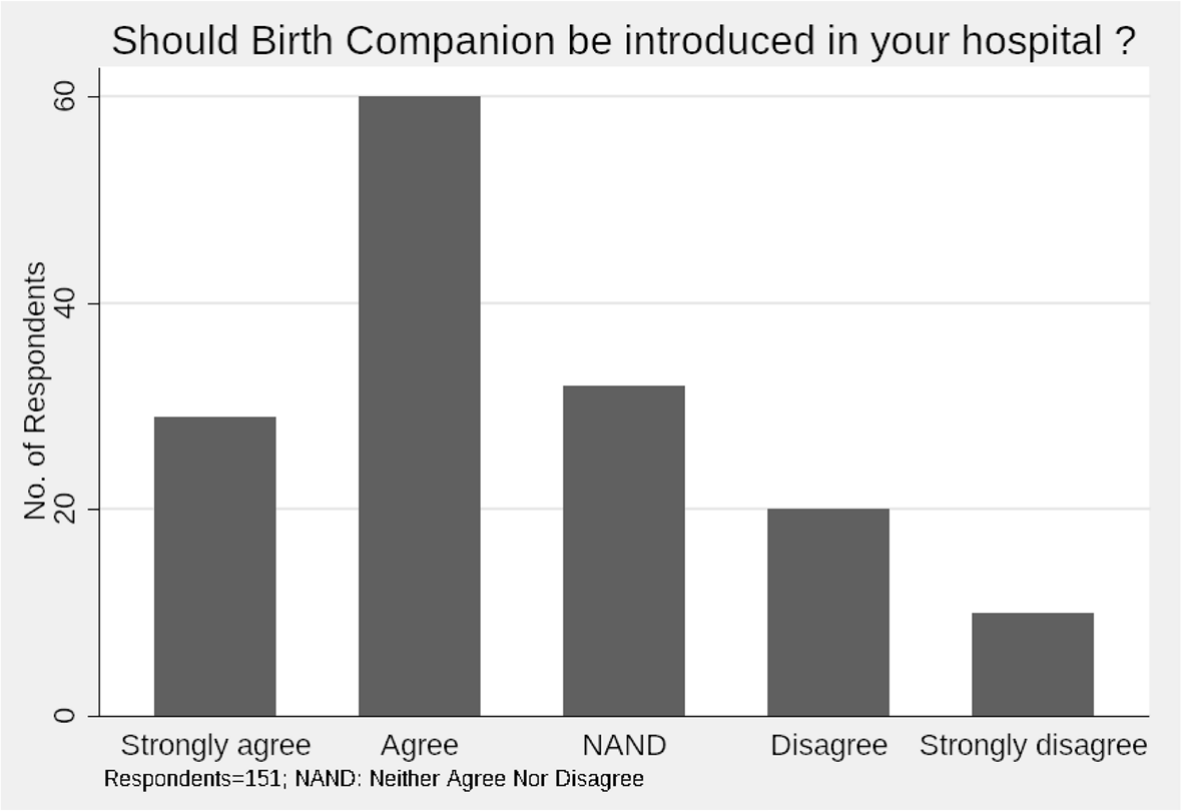
Response to Q12 of introducing birth companion

Seeking readiness for practice, respondents were asked if the concept of BC should be introduced in their institution. Only 19% of respondents strongly agreed while 40% agreed to this suggestion (Table 6, Fig. 1). The median response was “Agree”. This response was less favorable than to the question on the expected benefits of a BC where the overall perception was “highly beneficial”.

**Table 6:** Responses to Benefits of BC vs. introducing it in their Hospital

Q9. Presence of a BC Beneficial ?	Q.12. Should this concept be introduced in a tertiary care institution like MAMC					
	Strongly Agree	Agree	NAND	Disagree	Strongly Disagree	Total
	N					
	(Row, Column Percentages)					
Highly beneficial	24	34	10	8	1	77
	(31.17)	(44.16)	(12.99)	(10.39)	(1.3)	(100)
	(82.76)	(56.67)	(31.25)	(40)	(10)	(50.99)
Somewhat beneficial	5	25	21	10	6	67
	(7.46)	(37.31)	(31.34)	(14.93)	(8.96)	(100)
	(17.24)	(41.67)	(65.63)	(50)	(60)	(44.37)
Not beneficial	0	1	1	2	3	7
	(0)	(14.29)	(14.29)	(28.57)	(42.86)	(100)
	(0)	(1.67)	(3.13)	(10)	(30)	(4.64)
Total	29	60	32	20	10	151
	(19.21)	(39.74)	(21.19)	(13.25)	(6.62)	(100)
	(100)	(100)	(100)	(100)	(100)	(100)

Pearson chi2(8) = 38.9554 Pr = 0

Cramér's V = 0.3592

gamma = 0.565 ASE = 0.088

Kendall's tau-b = 0.3728 ASE = 0.064

Fisher's exact = 0

NAND: Neither Agree Nor Disagree

#### Comparison of perceived benefits versus readiness to introduction

Only 75% of the respondents who indicated that they thought BC as highly beneficial agreed to the suggestion of it being implemented in their hospital (Table 6). A smaller percentage of respondents (45%) who observed BC to be somewhat beneficial agreed to its introduction in their hospital (Table 6). These responses to the questions on expected benefits of BC and introducing it in their hospital were significantly related with a Pearson Chi Square statistic (χ2) with 8 degrees of freedom of 38.9 (*p*- value: 0.00), Cramér's V of 0.35, Kendall's tau-b of 0.37 (ASE: 0.064), Kruskal’s gamma statistic at 0.56 and ASE of 0.08 (Table 6).

### Barriers to BC perceived by healthcare providers

Key barriers to the introduction of a BC at the institution level included overcrowding in the labour room and privacy concerns for other women, especially in the presence of a male companion. Most respondents also “agreed” with the suggestion that hospital policy could be a barrier, and that BC posed a risk of infection; interference in clinical decisions, and issues of confidentiality. Personal barriers included embarrassment in giving birth in front of someone else, lack of access to a trustworthy person and economic loss due to the expense of hiring such a person.

### Suggestions from caregivers on overcoming barriers

On overcoming barriers to the introduction of BC, most respondents strongly agreed to the suggestions for increasing awareness among the hospital staff, providing funding to hospitals, creating physical partitions to ensure privacy, prior training of BC on their role, incentivizing women and hospitals, and formulation of guidelines.

**Fig. 2:**
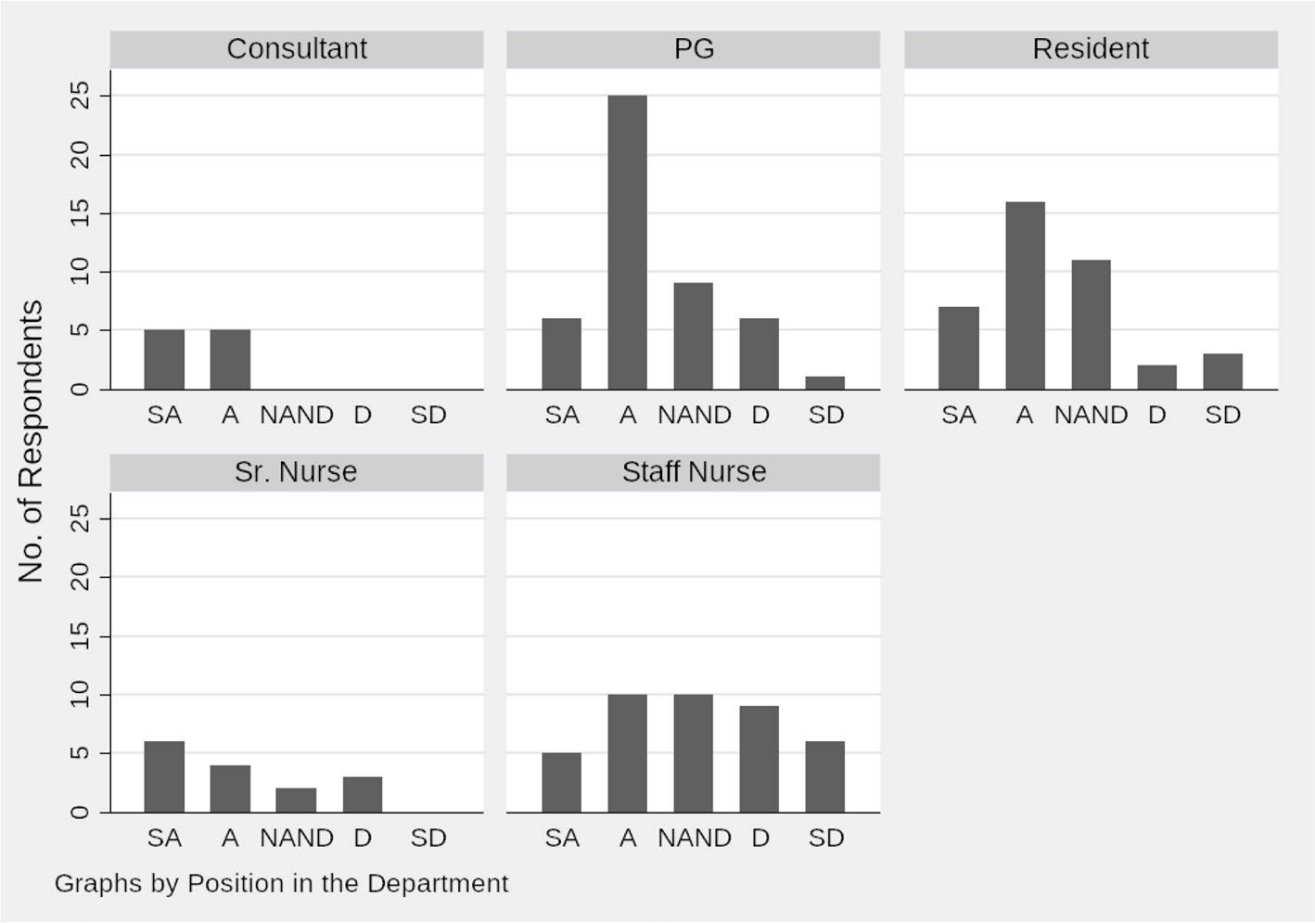
Histogram on whether BC Should be Introduced in their Hospital by Position of Responders

Consultants were the most supportive (Fig. 2) while staff nurses were the least approving of the idea of introducing BC in their institution. Kruskal–Wallis one-way analysis of variance to test the null hypothesis that the medians of all groups are equal was rejected with a Chi-Square value of 16.8 (p-0.001). Using quantile regression which estimates the median of the dependent variable, conditional on the values of the independent variable, we found that as we move lower in the healthcare worker hierarchy, the support for the introduction of BC in their hospital falls (coefficient: 0.33, t: 5.59, p: 0.00).

## Discussion

Our respondents covered the full spectrum of doctor and nurse healthcare providers who possessed a diverse experience in managing labour and childbirth in a large, tertiary care hospital with a high case load of childbirths. A consensus or broad agreement among such a broad group of care-givers signifies a widely held view and therefore carries a high degree of credibility, and generalizability.

With the advent of the COVID-19 pandemic, maternal and child health services have suffered a setback [44]. The mental health of pregnant women has also been affected [45]. BC can be utilized to minimize avoidable woman-clinician interface, optimize manpower at this critical time and help the birthing women cope with the dual challenge.

Thus, the possibility of improved maternal and neonatal outcomes by supporting the presence of a BC has immense public health importance, particularly in the face of COVID-19 pandemic.

Even though the choice remains with a birthing woman, caregivers in our study identified the mother and husband as the most preferred BC. These findings are similar to those of a study in Nigeria [46]. The Government of India in 2016 suggested that BC be a female relative, preferably one who has undergone the process of labor, be free of communicable diseases, wear clean clothes, not interfere in the work of hospital staff and not attend to other women [34].

Since women’s childbirth satisfaction is closely dependent on ensuring privacy [10], policy and systems for permitting BC should allow for sufficient privacy for other women giving birth in the same ward in the presence of a BC of any gender.

The prerequisites identified for a BC include basics like hygiene, being disease-free and their availability. While introducing the concept of BC, screening for communicable diseases and coaching on maintaining hygiene can be beneficial.

High levels of awareness of BC were matched among 95% of respondents to its expected benefits. Consultants perceived that BC was highly beneficial in high risk pregnancies. In our study, 70% of respondents felt that the presence of a BC would reduce the need for analgesia. Our findings are consistent with those from a study in Kenya​ [39] and Sri Lanka [23] as well as those from other studies​ [47] and a Cochrane review [17].

Despite fairly high levels of awareness, usefulness and perceived benefits of BC, only 61% of respondents who perceived BC to be beneficial agreed to its introduction in their hospital. Staff nurses were the least agreeable, with only 40% of those who agreed that BC were beneficial agreed to its introduction. Nurses as a group compared to doctors had a statistically significant disagreement in their response on introducing BC (Z = -2.183; *p* = 0.03). The reasons for this difference between awareness and willingness to practice, and among the classes of caregivers, points to practical difficulties in introducing BC. These difficulties are captured in response to the questions on barriers and how these can be overcome.

Respondents of all categories strongly agreed that the key barrier to introducing BC in their hospital was overcrowding and privacy concerns for other women. This is similar to other studies from Kenya ​[39] and Sri Lanka [23]. That lack of physical space leading to privacy issues was the key constraint in introducing BC is supported by funding for hospitals emerging as the most preferred response to the question on overcoming these barriers. Our findings are consistent with WHO’s recommendation on the need for implementing a strategy to sensitize health professionals, community and women towards the acceptance of the concept of BC [20, 39].

The strengths of this study are – universal total sampling with a relatively high response rate thus reducing selection bias; the limitation of our study is that its findings are only generalizable to tertiary care teaching institutions. Larger studies across institutions at all levels of healthcare in different socio-cultural settings are required.

In general, awareness among participants about the concept of BC was fairly high. Awareness, however, needs to be raised further.

There was a broad agreement that the concept of BC should be introduced in a tertiary care institution. For this to happen, barriers to implementation need to be identified, stakeholders engaged​ [48] and an action plan drawn and implemented.

Based on this study, we make the following recommendations:

1. The concept of BC should be part of the teaching curriculum for postgraduate medical and nursing students, which should be updated from time to time through Continuing Medical Education.
2. All cadres of staff working in labour rooms should be sensitized about the LaQshya program.
3. Pregnant women should be informed during antenatal visits of the benefits of a BC, and their right to have a BC of their choice.
4. A BC should be encouraged to accompany a pregnant woman for antenatal visits, who should be screened and counseled to prepare them for this role.
5. Infrastructure in health facilities should be strengthened so that it is conducive to the presence of BC. Available funds should be used to provide temporary partitions or curtains to create privacy for the women and their BC during birthing.
6. India’s prescribed National Quality Assurance Standards for Public Health facilities already requires (Standard B3) that the facility maintains the privacy, confidentiality & dignity of women. This needs to be implemented diligently. Hospitals that allow BC should be incentivized.
7. Key stakeholders need to be engaged while drawing the action plan, and for its effective implementation.

## Supplementary Information

**Additonal file 1.**

**Additional file 2.**

**Additional file 3.**

## Data Availability

All data generated or analyzed during this study are included in this published article and its supplementary information files.

## Abbreviations

BC: Birth Companion
LaQshya: Labour Room Quality Improvement Initiative
