# Supplementary material for: Healthcare providers perceptions regarding the presence of Birth Companion during childbirth at a tertiary care hospital in India
Source: BMC Pregnancy Childbirth. 2023 Mar 10;23:159. doi: 10.1186/s12884-022-05327-1 (PMC9999324; doi:10.1186/s12884-022-05327-1)
Supplement: Supplementary file 1 — Additonal file 1. [file 12884_2022_5327_MOESM1_ESM.docx]

**Annexure-1**

**Maulana Azad Medical College and Lok Nayak Hospital, New Delhi —110 002**

**(Department of Obstetrics & Gynecology)**

**STUDY PARTICIPANT INFORMATION SHEET**

You are being invited to participate in a research study.

Before you take part in this research study, the study will be explained to you and you will be given the chance to ask questions. Please read carefully the information provided here. If you agree to participate, please sign the Informed Consent Form. You will be given a copy of this document to take home with you.

**STUDY lNFORMATlON**

**Protocol Title:**

Awareness regarding, barriers to and suggestions for implementation of Birth Companion in labour and delivery: A cross sectional study among healthcare providers in a tertiary level teaching hospital in Delhi, India.

**Principal Investigator(s):**

……….., MBBS Student &

………..., Professor, MAMC.

**PURPOSE OF THE RESEARCH STUDY**

You are being invited to participate in a research study of Awareness regarding, Barriers to and Suggestions for allowing Birth companion during labour and delivery. We hope to learn the reasons for non adoption of Birth Companion in tertiary level teaching institutions in India. You were selected as a possible subject in this study because you are a part of the team of healthcare providers in Department of Obstetrics & Gynecology at a tertiary teaching hospital.

This study will recruit all the healthcare providers in Department of Obstetrics & Gynecology at Lok Nayak Hospital, which is a tertiary teaching hospital, for over a period of two months beginning June, 2019.

The study does not involve taking any sample of tissues, blood and/or body fluids.

The study does not involve or provide access to any study medication/device.

**STUDY PROCEDURES AND VISIT SCHEDULE**

If you agree to take part in this study, you will be asked to answer a questionnaire. Your participation in the study will last about 10 minutes. You will not need to visit any place other than your work place at any time in the course of the study.

**YOUR RESPONSIBILITIES IN THIS STUDY**

If you agree to participate in this study, you should:

- Answer the questionnaire for the study.

**WITHDRAWAL FROM STUDY**

You are free to withdraw your consent and discontinue your participation at any time without any prejudice to you. If you decide to stop taking part in this study, you should tell the Principal Investigator. There are no adverse effects to you from possible withdrawl from this study.

The Principal Investigator of this study may stop your participation in the study at any time for one or more of the following reasons:

- Failure to follow the instructions of the Principal Investigator.

- The study is canceled.

- Other administrative reasons.

- Unanticipated circumstances.

**WHAT IS NOT STANDARD CARE OR EXPERIMENTAL IN THIS STUDY**

The study does not involve testing or providing any care or investigation.

**POSSIBLE RISKS, DISCOMFORTS AND INCONVENIENCES**

There are no risks, discomforts or any inconveniences associated with this research study.

**POTENTIAL BENEFlTS**

If you participate in this study you may reasonably expect to benefit from this study by knowing more about the concept and benefits of Birth Companion during labour and delivery, which is an important component of the national Labour Room Quality Improvement Initiative.

In addition, your participation may contribute to the knowledge about the gaps in policy and guidelines of the national Labour Room Quality Improvement Initiative.

**SUBJECT’S RIGHTS**

Your participation in this study is entirely voluntary. Your questions will be answered clearly and to your satisfaction.

In the event of any new information becoming available that may be relevant to your willingness to continue in this study, you or your legal representative will be informed in a timely manner by the Principal Investigator or his/her representative.

By signing and participating in the study, you do not waive any of your legal rights to revoke your consent and withdraw from the study at any time.

**CONFIDENTIALITY OF STUDY AND MEDICAL RECORDS**

Information collected for this study will be kept confidential. Your records, to the extent of the applicable laws and regulations, will not be made publicly available. Only your investigator (s) will have access to the confidential information being collected.

By signing the Informed Consent Form attached, you or your legal representative is authorizing such access to your study records.

Data collected and entered into the Case Report Forms are the property of MAMC. In the event of any publication regarding this study, your identity will remain confidential.

**COSTS OF PARTICIPATION**

Other than 10 minutes of your time, there are no costs involved in your participation in this study. You will not receive any compensation for participating in this study.

**RESEARCH RELATED lNJURY AND COMPENSATION**

Since the study does not involve any intervention or test, there is no question of any research related injury or compensation.

**WHO TO CONTACT IF YOU HAVE QUESTIONS**

If you have questions about this research study and your rights you may contact the Principal Investigator – ……….. E-mail …. or .. Department of Obs. & Gynae, MAMC.
