# Supplementary material for: Healthcare providers perceptions regarding the presence of Birth Companion during childbirth at a tertiary care hospital in India
Source: BMC Pregnancy Childbirth. 2023 Mar 10;23:159. doi: 10.1186/s12884-022-05327-1 (PMC9999324; doi:10.1186/s12884-022-05327-1)
Supplement: Supplementary file 2 — Additional file 2. [file 12884_2022_5327_MOESM2_ESM.docx]

**Annexure-2**

| **CONSENT BY RESEARCH SUBJECT** |
| --- |

**Details of Research Study**

**Protocol Title:**

**Awareness regarding, barriers to and suggestions for implementation of Birth Companion in labour and delivery: A cross sectional study among healthcare providers in a tertiary level teaching hospital in Delhi, India.**

**Principal Investigator:**

…………………. MBBS Student &

…………………. Department of Obs. & Gynae, MAMC.

**Subject’s Particulars**

Name:

Sex: Female/Male

**Part I — to be filled by participant**

I__________________________ (Name of Participant) **agree / do not agree** to participate in the research study as described and on the terms set out in the Participant Information Sheet. The nature of my participation in the proposed research study has been explained to me in ____________________________ (Language dialect) by …………………….. (PI).

I have fully discussed and understood the purpose of this study. I have been given the Participant Information Sheet and the opportunity to ask questions about this study and have received satisfactory answers and information.

I understand that my participation is voluntary and that I am free to withdraw at any time, without giving any reasons and without any medical care being affected.

I also give permission for information in my questionnaire to be used for research. In any event of publication, I understand that this information will not bear my name or other identifiers and that due care will be taken to preserve the confidentiality of this information.

_____________ ___________________________

(Signature of Participant) Date of Signing

**Part IV— Investigator’s Statement**

l, the undersigned, certify to the best of my knowledge that the nature and purpose of study was fully explained and clearly understood before the study participant's signing this informed consent form.

| Name of Investigator  ……………………………. | Signature | Date |
| --- | --- | --- |
