# Supplementary material for: Healthcare providers perceptions regarding the presence of Birth Companion during childbirth at a tertiary care hospital in India
Source: BMC Pregnancy Childbirth. 2023 Mar 10;23:159. doi: 10.1186/s12884-022-05327-1 (PMC9999324; doi:10.1186/s12884-022-05327-1)
Supplement: Supplementary file 3 — Additional file 3. [file 12884_2022_5327_MOESM3_ESM.docx]

**Annexure-3**

**Questionnaire for Assessing Awareness regarding, barriers to and suggestions for implementation of Birth Companion in labour and delivery**

*Required

1. Name

2. Age

3a. Gender *

- Male
- Female

3b. Position in Department of Obstetrics and Gynecology, MAMC / Lok Nayak Hospital:

- Consultant
- Resident
- Post Graduate (PG)
- Senior Nurse
- Staff Nurse

4. Are you aware of the concept of Birth Companion?

- Fully Aware
- Somewhat Aware
- Not Aware

5. Are you aware that the WHO recommends every woman to be accompanied by a companion of her choice during labour?

- Fully Aware
- Somewhat Aware
- Not aware

6. Are you aware that the Government of India has recently advocated the presence of a birth companion in all Government hospitals?

- Fully Aware
- Somewhat Aware
- Not Aware

7. Who in your opinion can qualify to be a birth companion? (More than one answer is allowed)

- Husband / Spouse
- Mother
- Sister
- Mother-in-law
- Children
- Friend
- Daula
- Doctor
- Nurse

8. Pre-requisites for a birth companion?

8.1 Should be a female relative.

- True
- False

8.2 She should have gone through process of labour

- True
- False

8.3 Should not suffer from any communicable disease

- True
- False

8.4 She should wear clean clothes

- True
- False

8.5 She should stay with the pregnant woman throughout the process of labour

- True
- False

8.6 She should interfere with the work of hospital staff

- True
- False

8.7 She should attend to other women in the labour room

- True
- False

9. In your opinion, is presence of a birth companion during labour beneficial?

- Highly beneficial
- Somewhat beneficial
- Not beneficial

10. If yes, what in your opinion are the benefits of a birth companion?

10.1 Shorter duration of labour

- True
- False

10.2 Reduced need for analgesia

- True
- False

10.3 Increased spontaneous vaginal births

- True
- False

10.4 Reduced need for instrumental deliveries

- True
- False

10.5 Reduced incidence of unnecessary caesarian sections

- True
- False

10.6 Humanization of labour

- True
- False

10.7 Reduced workload for hospital staff

- True
- False

10.8 Avoid frequent vaginal examinations

- True
- False

10.9 Reduced intra-partum bleeding

- True
- False

10.10 Provide emotional support

- True
- False

10.11 Provide comfort measures – soothing touch, massage

- True
- False

10.12 Help the woman to advocate her wish to others

- True
- False

10.13 Help in delivering in the birth position of choice

- True
- False

10.14 Boost the woman’s confidence

- True
- False

10.15 Provide spiritual support

- True
- False

10.16 Increased satisfaction by the women in labour

- True
- False

10.17 Increased use of partograph

- True
- False

10.18 Help in early initiation of breastfeeding

- True
- False

10.19 Higher newborn Apgar score

- True
- False

10.20 Reduce post-partum depression

- True
- False

11. Will the concept of birth companion be beneficial in dealing with high risk pregnancies?

- Highly beneficial
- Somewhat beneficial
- Not beneficial

12. Should this concept be introduced in a tertiary care institution like MAMC & Lok Nayak Hospital?

- Strongly agree
- Agree
- Neither agree nor disagree
- Disagree
- Strongly disagree

13. What according to you are the barriers to implementation of the concept of birth companion in this institution?

13.1 At the Institution Level:

13.1.1 Hospital policy

- Strongly agree
- Agree
- Neither agree not disagree
- Disagree
- Strongly disagree

13.1.2 Overcrowding in the labour room

- Strongly agree
- Agree
- Neither agree not disagree
- Disagree
- Strongly disagree

13.1.3 Lack of privacy for the woman (no dividers or curtains)

- Strongly agree
- Agree
- Neither agree not disagree
- Disagree
- Strongly disagree

13.1.4 Privacy concerns for other women, especially in presence of a male companion

- Strongly agree
- Agree
- Neither agree not disagree
- Disagree
- Strongly disagree

13.1.5 Risk of infection transmission

- Strongly agree
- Agree
- Neither agree not disagree
- Disagree
- Strongly disagree

13.1.6 Asepsis in the operation theatre (OT) will be compromised

- Strongly agree
- Agree
- Neither agree not disagree
- Disagree
- Strongly disagree

13.1.7 Women would not cooperate with hospital staff in presence of birth companion

- Strongly agree
- Agree
- Neither agree not disagree
- Disagree
- Strongly disagree

13.1.8 Use of traditional medicines by the birth companion

- Strongly agree
- Agree
- Neither agree not disagree
- Disagree
- Strongly disagree

13.1.9 Birth companion may interfere with clinical decision making

- Strongly agree
- Agree
- Neither agree not disagree
- Disagree
- Strongly disagree

13.1.10 A lounge / waiting area will be needed for the birth companion to take short breaks

- Strongly agree
- Agree
- Neither agree not disagree
- Disagree
- Strongly disagree

13.1.11 The birth companion may not be continuously present during labour

- Strongly agree
- Agree
- Neither agree not disagree
- Disagree
- Strongly disagree

13.1.12 Birth companion will not be able to face the sight of blood

- Strongly agree
- Agree
- Neither agree not disagree
- Disagree
- Strongly disagree

13.1.13 Woman’s health information should be kept confidential

- Strongly agree
- Agree
- Neither agree not disagree
- Disagree
- Strongly disagree

13.1.14 Presence of birth companion is unprofessional

- Strongly agree
- Agree
- Neither agree not disagree
- Disagree
- Strongly disagree

13.2 For the patient:

13.2.1 Embarrassment of delivering in front of someone else

- Strongly agree
- Agree
- Neither agree not disagree
- Disagree
- Strongly disagree

13.2.2 Not socially acceptable (stigma / customs)

- Strongly agree
- Agree
- Neither agree not disagree
- Disagree
- Strongly disagree

13.2.3 Birth companion may gossip in the community

- Strongly agree
- Agree
- Neither agree not disagree
- Disagree
- Strongly disagree

13.2.4 Cost of transportation of the birth companion to the hospital

- Strongly agree
- Agree
- Neither agree not disagree
- Disagree
- Strongly disagree

13.2.5 Economic loss if the birth companion is an earning member and has to take break from work

- Strongly agree
- Agree
- Neither agree not disagree
- Disagree
- Strongly disagree

13.2.6 Hiring a doula is expensive

- Strongly agree
- Agree
- Neither agree not disagree
- Disagree
- Strongly disagree

13.2.7 No trustworthy person to accompany her as a birth companion

- Strongly agree
- Agree
- Neither agree not disagree
- Disagree
- Strongly disagree

14. How can these barriers be overcome?

14.1 Increased awareness among the hospital staff about the benefits of birth companions

- Strongly agree
- Agree
- Neither agree not disagree
- Disagree
- Strongly disagree

14.2 Creating physical partitions to ensure privacy

- Strongly agree
- Agree
- Neither agree not disagree
- Disagree
- Strongly disagree

14.3 Providing incentives to women delivering in the presence of a birth companion

- Strongly agree
- Agree
- Neither agree not disagree
- Disagree
- Strongly disagree

14.4 Prior training of birth companions on their role

- Strongly agree
- Agree
- Neither agree not disagree
- Disagree
- Strongly disagree

15.1 Provide funding to hospitals to upgrade labour rooms

- Strongly agree
- Agree
- Neither agree not disagree
- Disagree
- Strongly disagree

15.2 Incentivize hospitals that allow birth companions

- Strongly agree
- Agree
- Neither agree not disagree
- Disagree
- Strongly disagree

15.3 Formulate guidelines for instructing birth companions

- Strongly agree
- Agree
- Neither agree not disagree
- Disagree
- Strongly disagree

THANK YOU FOR YOUR TIME

(<https://docs.google.com/forms/d/e/1FAIpQLSd838qwowPfn2mUUdc_ut1x81kCGsC9nmgHbzY4yU3JYw-FTQ/viewform?vc=0&c=0&w=1>)
